# Cholesterol-cholate-butterfat diet offers multi-organ dysfunction in rats

**DOI:** 10.1186/1476-511X-13-194

**Published:** 2014-12-16

**Authors:** Humaira Jamshed, Jamshed Arslan, Anwar-ul-Hassan Gilani

**Affiliations:** Natural Product Research Unit, Department of Biological and Biomedical Sciences, Aga Khan University, Karachi, 74800 Pakistan; College of Health Sciences, Mekelle University, P. O. Box 1871, Mekelle, Ethiopia

**Keywords:** Fasting, Liver function, Vascular function, Memory

## Abstract

**Background:**

Comparable to commercial expensive high-fat diets, cholesterol-cholate-butterfat (CCB) diet has also been used to induce hyperlipidemia in rats. Our objective was to explore its influence on multiple organs. Consequence of fasting was also analysed.

**Methods:**

Rats in groups 1 and 2 received normal diet (ND) whereas groups 3 and 4 received CCB-diet. Food was withdrawn daily for two hours from groups 2 (ND-F) and 4 (CCB-F). Blood was collected at fourth and sixth week for biochemical estimation; Morris water maze was done in the sixth week for learning ability and memory; after which aortae were isolated for vascular reactivity.

**Results:**

Apart from hyperlipidemia, CCB also induced hyperglycemia with marked increase in hepatic enzymes: gamma-glutamyl transferase (GGT), alanine and aspartate aminotransferase (ALT and AST); and vascular biomarkers: uric acid (UA), phosphorus and alkaline phosphatase (ALP). Isolated aortae, pre-contracted with phenylephrine, were less responsive to acetylcholine indicating endothelial dysfunction – serum nitric oxide (NO) production was limited with subsequent inhibition of endothelial NO synthase. CCB diet also compromised learning ability. CCB-coupled fasting potentiated hyperlipidemia but prevented memory-loss.

**Conclusion:**

We introduce CCB-diet for multi-organ dysfunction in rats, and propose its use for research on cardiovascular diseases and associated manifestations involving immense interplay of integrated pathways.

**Electronic supplementary material:**

The online version of this article (doi:10.1186/1476-511X-13-194) contains supplementary material, which is available to authorized users.

## Background

Human diseases are complex – representing interplay of synchronized abnormalities in multiple organs. Cardiovascular diseases (CVDs) are the largest death burden globally [1]. They involve not only heart and vessels, but liver [2], kidneys [3] and even the nervous system [4]. As a result, common co-morbidities of CVDs are non-alcoholic fatty liver disease [5], chronic kidney disease [6] and Alzheimer’s disease etc. [7, 8]. Consequently, research on novel therapeutic interventions, also mandates a holistic approach, such that safety and efficacy is assessed on multiple systems simultaneously.

Animal models are excellent tools for such research, and aid in pathophysiological understanding of human ailments [9]. Genetically manipulated animals – although preferable for being precise [10, 11] – may not truly represent disorders as complex as CVDs. Alternatively, there are modified diets, inducing human-like pathologies in laboratory animals [12]. Food markedly impacts health. It influences disease status of humans [13] and animals [14]. Ingredients like fats are known to increase CVD risk factors in species like rabbits [15], hamsters [16], rats [14] and mice [17]. In laboratory animals, the alterations convened by high-fat diets (HFDs) are fairly similar to human [18]. Literature reports that commercial HFDs cause hyperlipidemia [19], which consequence in lipids’ deposition in tissues (both adipose and non-adipose). Eventually lipid build-up leads to cellular dysfunction of heart, vessels and liver [20].

Experimental manipulation of these pre-formed commercial diets could be challenging. In contrast, a simple modifiable diet containing cholesterol, cholate and butterfat (CCB) as fat sources, has also been used to induce hyperlipidemia [21]. We aim to inspect the possible influence of this CCB-diet on hepatic and vascular function along with learning ability in rats. These aspects have not yet been explored for CCB-diet. In addition, since dietary restriction is known to induce adaptive changes in intermediary metabolism [22], we also inquired the consequence of daily two-hour fasting.

## Results

### Lipid profile

**Figure 1:**
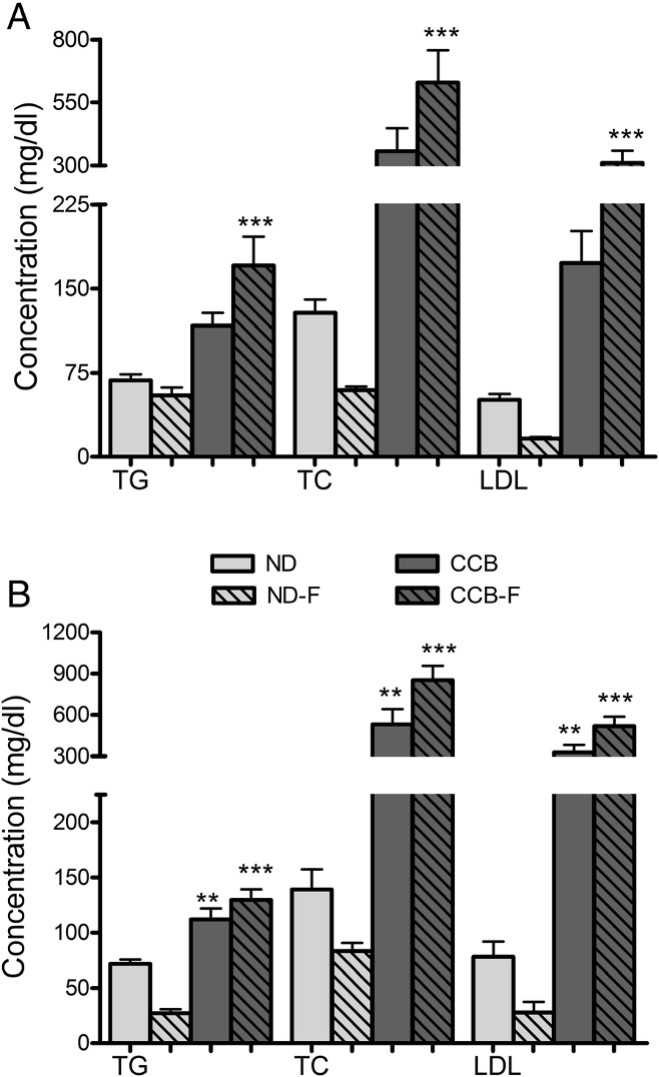
**CCB-diet alone and coupled with fasting, induced hyperlipidemia in rats. A)** Fourth week; **B)** Sixth week; TG: triglyceride; TC: total cholesterol; LDL: low-density lipoprotein; ND: normal diet; ND-F normal diet with two-hour fasting; CCB: cholesterol-cholate-butterfat diet; CCB-F: cholesterol-cholate-butterfat diet with two-hour fasting. All values are represented as mean ± standard error of mean (SEM) (n = 7 per group). This figure only shows the comparison of means using one-way ANOVA followed by Tuckey’s post-test (**p < 0.01 and ***p < 0.001).

CCB-diet caused pronounced hyperlipidemia at fourth and sixth week. Serum concentrations of TG, TC and LDL were drastically increased at fourth week, from 68 ± 5, 128 ± 12 and 51 ± 5 in ND to 117 ± 11, 430 ± 113 and 173 ± 29 mg/dl in CCB group (Figure 1A). Also at sixth week, as shown in figure 1B, the serum concentrations of TG, TC and LDL were markedly increase from 72 ± 4, 139 ± 19 and 78 ± 14 mg/dl in ND to 112 ± 10, 583 ± 119 and 327 ± 55 mg/dl in CCB respectively.

CCB coupled with fasting potentiated hyperlipidemia at fourth and sixth week, as evident from Figure 1A and B respectively. The resultant serum concentrations of TG, TC and LDL were respectively 170.6 ± 26, 629 ± 129 and 311 ± 49 mg/dl at fourth week and 130 ± 10, 853 ± 104 and 518 ± 68 mg/dl at sixth week.

For the effect of fasting *per se,* normal diet-fed rats were also fasted. Reduction in TC and LDL was observed at fourth and sixth week; TG was reduced at sixth week. Resultant concentrations of TC and LDL were respectively 59.7 ± 3.2 and 16.3 ± 1.3 mg/dl at fourth week and 83 ± 7.5 and 28 ± 9.8 mg/dl at sixth week; with TG concentration of 27 ± 3.4 mg/dl at sixth week.

### Lipid ratios

**Figure 2:**
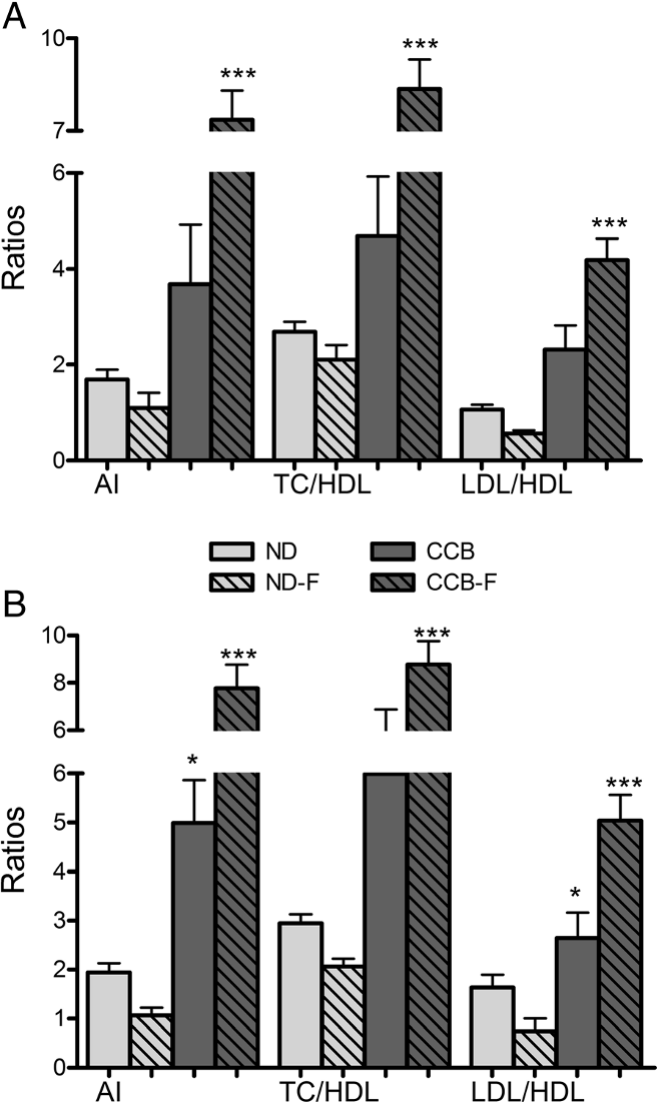
**CCB-diet alone and coupled with fasting, increased atherogenic index and lipid ratios. A)** Fourth week; **B)** Sixth week; AI: atherogenic index; ND: normal diet; ND-F normal diet with two-hour fasting; CCB: cholesterol-cholate-butterfat diet; CCB-F: cholesterol-cholate-butterfat diet with two-hour fasting. All values are represented as mean ± standard error of mean (SEM) (n = 7 per group). This figure only shows the comparison of means using one-way ANOVA followed by Tuckey’s post-test (*p < 0.05 and ***p < 0.001).

Results for the calculated lipid parameters show a noticeable increase (p < 0.01) in LDL/HDL ratio at fourth week, whereas, atherogenic index (AI) and TC/HDL ratios were elevated (p < 0.01) after six weeks (Figure 2A and B). Value of LDL/HDL was 1.06 ± 0.09 in ND and 2.78 ± 0.59 in CCB; atherogenic index and TC/HDL ratios at sixth week were respectively 1.94 ± 0.18 and 2.94 ± 0.18 in ND with 4.99 ± 0.88 and 5.99 ± 0.88 in CCB.

Fasting with CCB-diet potentiated the elevation of these ratios. The resultant values of AI, TC/HDL and LDL/HDL ratios were 7.4 ± 0.9, 8.4 ± 0.9 and 4.2 ± 0.4 at fourth week, and 7.8 ± 0.9, 8.8 ± 0.9 and 5 ± 0.5 at sixth week respectively (Figure 2A and B). In contrast, fasting with normal diet reduced LDL/HDL ratio (at fourth and sixth week) and AI and TC/HDL (at sixth week).

### Glucose and GGT

**Figure 3:**
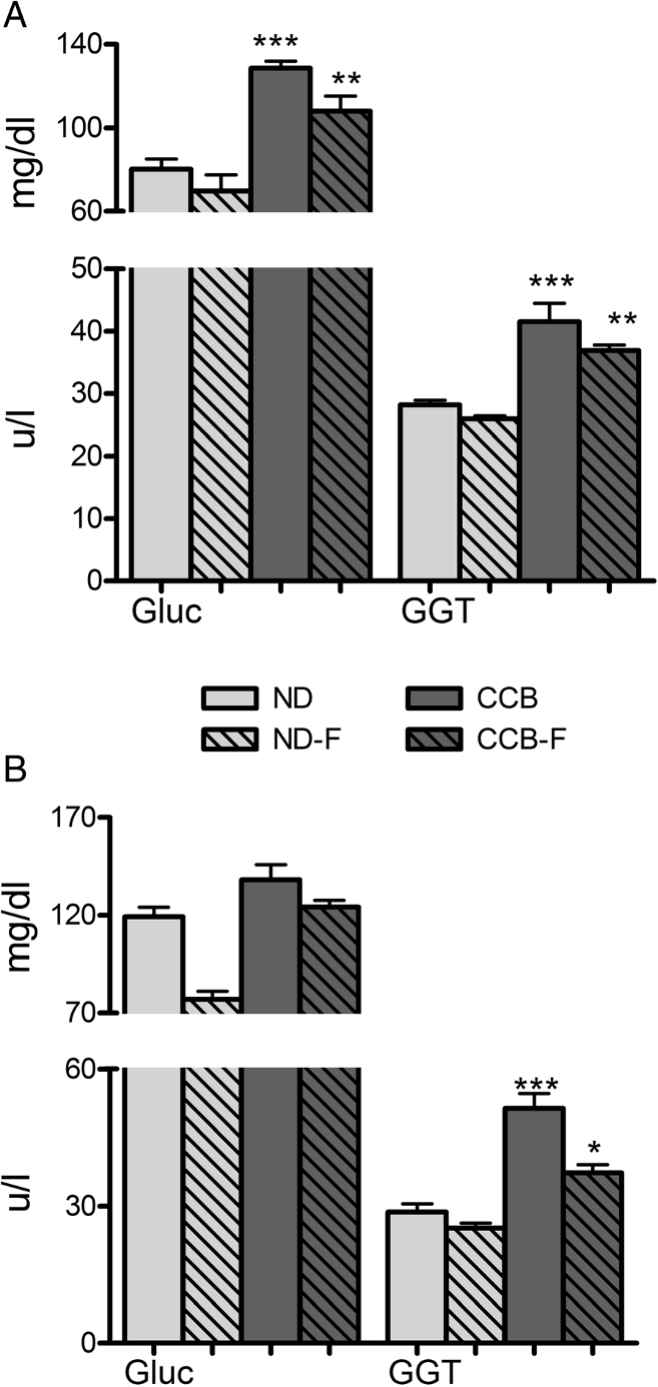
**CCB-diet alone or coupled with fasting, increased serum glucose and GGT in rats. A)** Fourth week; **B)** Sixth week; Gluc: glucose (mg/dl); GGT: gamma-glutamyl transferase (u/l); ND: normal diet; ND-F normal diet with two-hour fasting; CCB: cholesterol-cholate-butterfat diet; CCB-F: cholesterol-cholate-butterfat diet with two-hour fasting. All values are represented as mean ± standard error of mean (SEM) (n = 7 per group). This figure only shows the comparison of means using one-way ANOVA followed by Tuckey’s post-test (*p < 0.05, **p < 0.01 and ***p < 0.001).

Our data presented in Figure 3 show a prominent elevation (p < 0.001), by CCB-diet, in serum glucose (only at fourth week) and GGT (at fourth and sixth week). Consequent concentrations of glucose were 80 ± 5 mg/dl in ND and 129 ± 3 mg/dl in CCB. Serum GGT concentrations were 28 ± 0.7 u/l in ND and 41 ± 3 u/l in CCB (at fourth week) and 25.8 ± 33 u/l in ND and 43 ± 58 in CCB (at sixth week).

Glucose and GGT responded similarly when fasting was coupled with CCB, but when coupled with normal diet, fasting reduced serum glucose (to 69 ± 87 mg/dl), at sixth week.

### Hepatic function biomarkers

**Figure 4:**
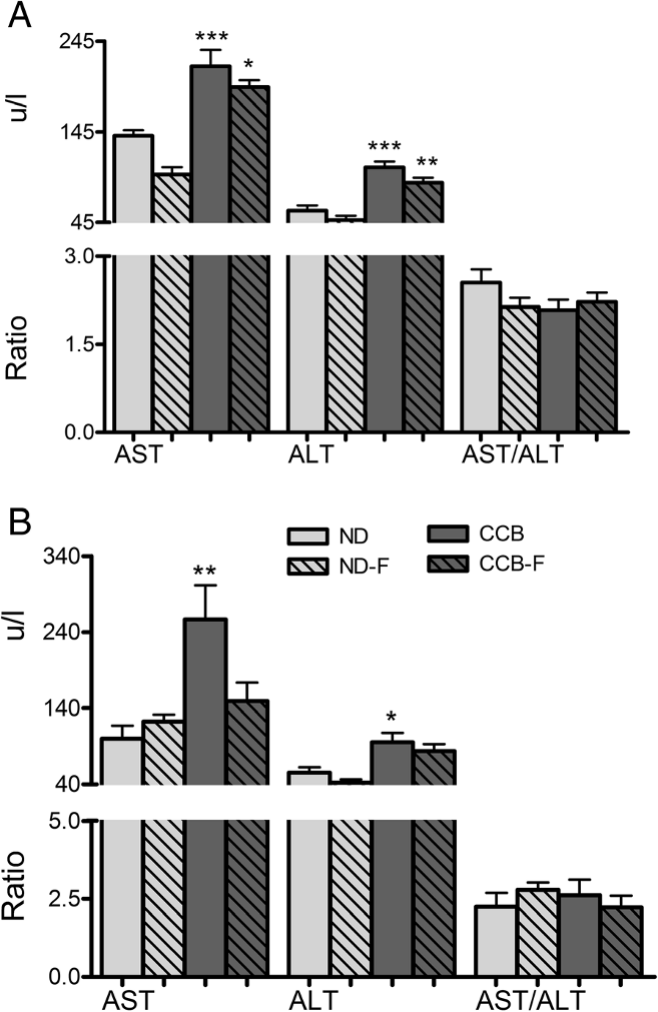
**CCB-diet alone or coupled with fasting, induced mild hepatic injury raising serum biomarkers. A)** Fourth week; **B)** Sixth week; AST: aspartate aminotransferase (u/l); ALT: alanine aminotransferase (u/l); ND: normal diet; ND-F normal diet with two-hour fasting; CCB: cholesterol-cholate-butterfat diet; CCB-F: cholesterol-cholate-butterfat diet with two-hour fasting. All values are represented as mean ± standard error of mean (SEM) (n = 7 per group). This figure only shows the comparison of means using one-way ANOVA followed by Tuckey’s post-test (*p < 0.05, **p < 0.01 and ***p < 0.001).

Other than GGT, indicators of hepatic function e.g. aminotransferases (AST and ALT) were also distinctly elevated by CCB-diet at fourth and sixth week as presented in Figure 4A and B respectively. At fourth week increase in AST and ALT were respectively 1.55 and 1.8 folds whereas at sixth week the concentrations were 2.56 and 1.7 folds higher in CCB compared to ND.

Two-hour fasting with CCB led to almost equivalent increase in these aminotransferases (p > 0.05) as CCB alone did, whereas fasting with normal diet decreased AST at fourth week. The calculated AST/ALT ratio remained unchanged in all groups throughout the experiment duration.

### Vascular function

**Figure 5:**
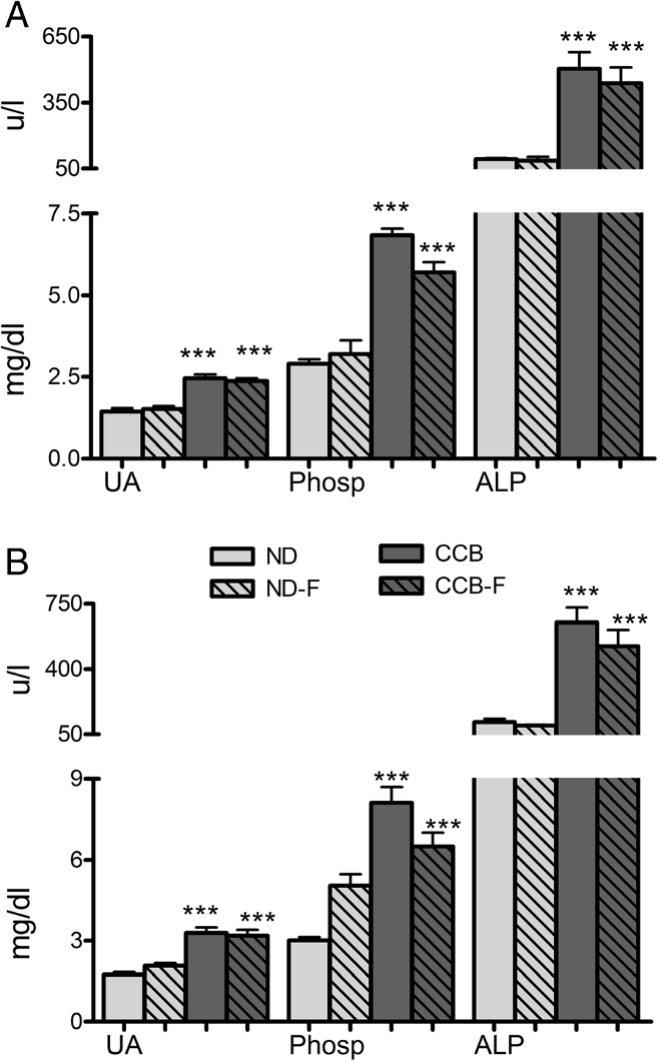
**CCB-diet alone and coupled with fasting, elevated vascular biomarkers in rats. A)** Fourth week; **B)** Sixth week; UA: uric acid (mg/dl); Phosp: phosphorus (mg/dl); ALP: alkaline phosphatase (u/l); ND: normal diet; ND-F normal diet with two-hour fasting; CCB: cholesterol-cholate-butterfat diet; CCB-F: cholesterol-cholate-butterfat diet with two-hour fasting. All values are represented as mean ± standard error of mean (SEM) (n = 7 per group). This figure only shows the comparison of means using one-way ANOVA followed by Tuckey’s post-test (***p < 0.001).

Vascular function was studied at three levels. Firstly, some bio-molecules associated with endothelial dysfunction (UA, phosphorus and ALP) were found to be elevated profoundly (p < 0.001) by CCB-diet. Fasting coupled with normal diet had no effect on these parameters (p > 0.05). Serum concentrations of UA were 1.4 ± 0.1, 2.4 ± 0.1 and 2.37 ± 0.08 mg/dl in ND, CCB and CCB-fasting respectively at fourth week and 1.7 ± 0.09, 3.29 ± 0.2 and 3.18 ± 0.2 mg/dl respectively at sixth week (Figure 5A and B). Phosphorus and ALP levels, at fourth week, were 2.9 ± 0.1, 6.8 ± 0.2 and 5.7 ± 0.3 mg/dl in ND, CCB and CCB-F, and 93 ± 4.7, 504 ± 76 and 437 ± 72 u/l respectively (Figure 5A). Whereas at sixth week, phosphorus was 3 ± 0.1, 8 ± 0.6 and 6.5 ± 0.5 mg/dl and ALP were 116 ± 17, 650 ± 79 and 522 ± 87 u/l respectively (Figure 5B).

**Figure 6:**
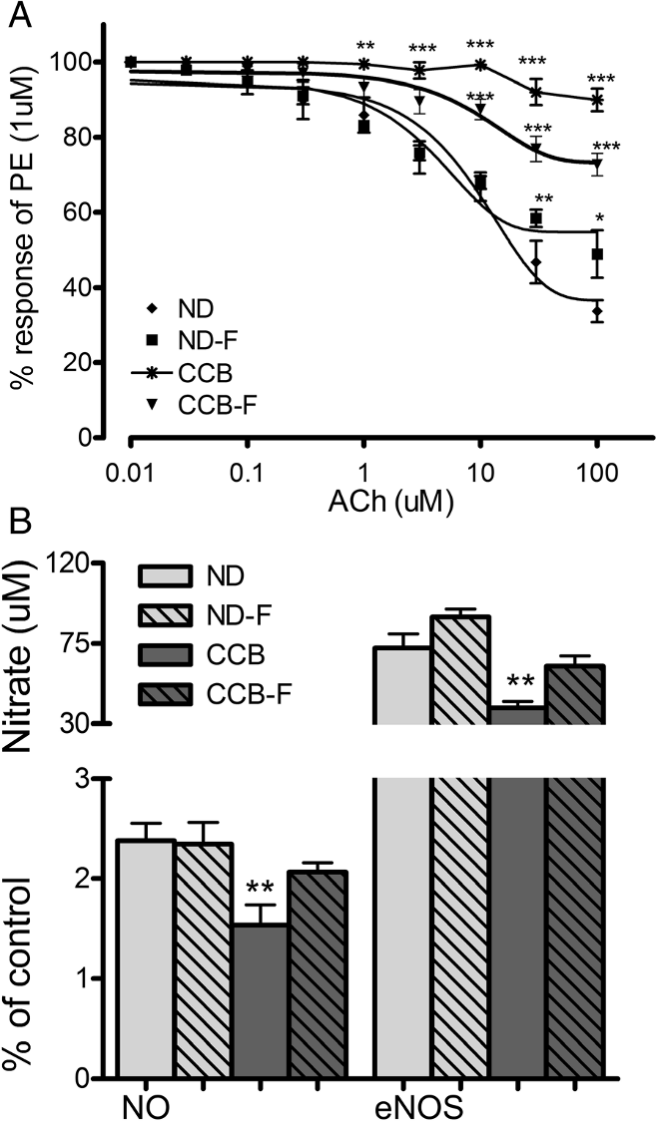
**CCB-diet impaired vascular reactivity of thoracic aortae by amending nitric oxide pathway. A)** Isolated aorta experiment; PE: phenylephrine; Ach: acetyl choline, Two-way ANOVA followed by Bonferroni’s post-test is applied. **B)** Nitric oxide pathway; NO: nitric oxide (uM); eNOS: endothelial nitric oxide synthase; ND: normal diet; ND-F normal diet with two-hour fasting; CCB: cholesterol-cholate-butterfat diet; CCB-F: cholesterol-cholate-butterfat diet with two-hour fasting. All values are represented as mean ± standard error of mean (SEM) (n = 7 per group). This figure only shows the comparison of means using one-way ANOVA followed by Tuckey’s post-test (*p < 0.05, **p < 0.01 and ***p < 0.001).

In the second step, vascular function of isolated thoracic aortae was analysed on isolated tissue bath assembly. Concentration-response curves of acetylcholine (ACh: 0.01 μM to 100 μM) were prepared after pre-contracting the aortae with phenylephrine (1 × 10^−6^ mol/L). As shown in figure 6A, acetylcholine (at concentration of 1 μM and above) inhibited the phenylephrine-induced contractions of aortic rings from normal controls in a concentration-dependent manner. However, in aortae from CCB-fed rats, this response was inhibited (p < 0.001) at high concentrations of ACh (i.e. 3 μM and above), indicating endothelial dysfunction. Fasting coupled with CCB partly prevented this impairment, evident in Figure 6A by the partial inhibition of PE-induced contraction by ACh at high concentrations (10 μM and above).

Thirdly, we explored the probable downstream events contributing to endothelial dysfunction. Endothelial nitric oxide synthase (eNOS) activity was assayed in aorta, and total nitric oxide (NO) concentration was measured in serum. As elaborated in Figure 6B, production of serum NO was diminished perhaps due to inactivation of eNOS by the CCB-diet. Enzyme activity (represented as % of control) and nitrate concentrations in ND were 72.5 ± 7.9% of control and 2.4 ± 0.17 uM respectively. In CCB, eNOS and nitrate were 38.7 ± 3.7% of control and 1.5 ± 0.2 uM respectively (p < 0.01). In fasting with CCB, the concentrations of eNOS and NO were 62 ± 5.9% of control and 2 ± 0.9 uM respectively. Fasting almost completely protected the CCB-induced endothelial dysfunction such that eNOS activity and NO concentration were similar to normal controls (p > 0.05).

### Learning/memory

**Figure 7:**
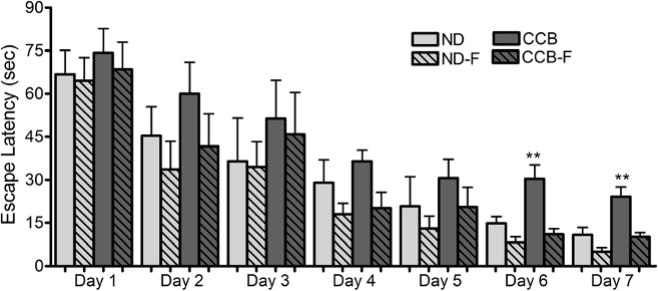
**CCB-diet impaired learning ability in rats.** ND: normal diet; ND-F normal diet with two-hour fasting; CCB: cholesterol-cholate-butterfat diet; CCB-F: cholesterol-cholate-butterfat diet with two-hour fasting. All values are represented as mean ± standard error of mean (SEM) (n = 7 per group). This figure only shows the comparison of means using one-way ANOVA followed by Tuckey’s post-test (**p < 0.01).

The CCB-diet slowed the learning process and/or impaired memory. This was evident on day six and day seven of Moris water maze (MWM), when CCB-fed rats took longer time (p < 0.01) to find the hidden platform (escape latency), compared to normal controls. On the first five days of MWM trials, there was no significant difference in the escape latency of rats from different groups, as evident in Figure 7. Escape latency on day six and seven were 2.05 and 2.24 times higher in CCB compared to ND (Figure 7). Fasting had no significant effect on normal controls, but almost completely prevented CCB-induced memory loss, as response was similar to normal controls.

## Discussion

Our study presents CCB-diet model as a candidate for research on cardiovascular diseases with associated complications, as this diet ensures the multi-organ dysfunction induced in rats. In addition to serum lipid profile and glucose, interestingly, this CCB-diet also elevated biomarkers of hepatic and vascular function. CCB impaired the vascular reactivity of isolated aorta, by restraining eNOS activity, thereby limiting nitric oxide production. Besides, this simple high-fat diet containing cholesterol, cholate and butterfat, also affected the nervous system by lessening the learning capabilities of experimental rats.

Comparable to other commercial HFDs [20], CCB-diet increased triglyceride, cholesterol and LDL, with little effect on HDL. The lipid profile is considered as a good indicator of cardiovascular health status. Nevertheless, it is suitable mainly for extreme high and extreme low risk individuals and not the majority at medium risk [23]. Conversely, TC/HDL and LDL/HDL ratios have been shown to be better markers than LDL and HDL levels per se [24–26]. TC/HDL is known to be a better predictor of ischemic heart disease than LDL/HDL ratio [27] since it involves very-low-density-lipoprotein (VLDL) and intermediate-density-lipoprotein, in addition to LDL and HDL. Although the significance of LDL/HDL ratio is believed to be compromised in hypertriglyceridemias (where majority of the serum cholesterol resides in VLDL); yet it is an excellent indicator of drug response [28] as it simultaneously represents atherogenic and anti-atherogenic lipids. This is why we calculated and presented the results of these ratios.

The hypertriglyceridemia and LDL elevation we obtained could be attributable to the cholesterol in CCB-diet: it accelerates glycolysis in liver [29], producing fatty acids which are esterified to triglycerides and cholesterol-esters [30]. These esters integrate in LDL, and release in blood [31], along with the excess triglycerides [32]. Dietary cholesterol also blocks the receptor-mediated LDL uptake sustaining increased plasma LDL [33]. While explaining CCB-diet induced hypercholesterolemia, however, we do realize that humans and rats resist dietary cholesterol-induced hypercholesterolemia [34] in contrast to rabbits and hamsters [35, 36]. Whereby, cholesterol blocks the de-novo synthesis by inhibiting HMG Co-A reductase [37], facilitates the catabolic conversion of cholesterol to bile acids [38] and accelerates biliary cholesterol excretion [39]. This prevents the rise in serum cholesterol, where bile overproduction ensures that excess cholesterol is eliminated.

The butterfat in CCB-diet contains palmitic, oleic and stearic acids. It counteracts by enhancing de-novo cholesterol synthesis via activation of HMG Co-A reductase [40, 41]. Our CCB-diet is also supplemented with cholate, which ensures greater intestinal cholesterol absorption [42] in addition to promoting cholesterol synthesis [43]. The probable synergism of butterfat and cholate could justify hypercholesterolemia in the CCB-diet model.

Our results showed that two-hour fasting promoted CCB-induced hyperlipidemia. Body adopt dietary amendments by altering metabolism. In well-fed state, energy is provided by carbohydrate and sugars, which steadily shifts to fats in case of fasting [44]. The fat stores (in adipocytes) disintegrate, supplying fatty acids to liver [45], increasing the cholesterol content - which secretes in blood as LDL after esterification [46]. Hepatic fatty acids should esterify causing hypertriglyceridemia [47] but we observed similar triglycerides in CCB and CCB-fasting (at sixth week). This may be because, hepatic fatty acid mainly arises from adipose stores with minor amounts synthesized from dietary sources [48]. In case of a chronic fasting (six weeks), these stores exhausted and were probably no longer accessible. Prolonged fasting also inhibits fatty acid synthesis and promotes its oxidation [49]. Fasting coupled with normal diet also reduced triglycerides. This might be because ND was not supplemented with additional fatty acids. In absence of exogenous source, depletion of endogenous stores and accelerated oxidation of fatty acids – triglycerides decreased. Extended use of CCB-diet supplemented with fatty acids (in butterfat), prevented the fall in TG.

Al-Attar (2010) has reported the combined effects of intermittent fasting and high-fat diet [22]. Where we have used cholesterol, cholate and butterfat as the source of high fat in diet, Al-Attar (2010) used 15% mutton tallow (and the remaining diet composition was not provided). This might underlie the contradicting results; they obtained similar hyperlipidemia by high-fat diet (HFD) with and without fasting. The fasting duration also varied, from two hours daily in our investigation, to 10 hours/day for five days a week in the study by Al-Attar (2010).

In addition to hyperlipidemia, we found elevation in GGT, AST and ALT by the CCB-diet. Apart from the general perception of GGT as an indicator of hepatic injury, it is now also recognized as a predictor of cardiovascular event [50]. GGT is basically a marker of oxidative stress [51] and inflammation [52], and is linked with hypertension [53, 54] and hyperlipidemia [55]. Besides, it is associated with CVD [56] and reported to be present in atherosclerotic plaques [57, 58], where it is anticipated to be involved in LDL oxidation [59]. Likewise, the aminotransferases (both ALT and AST) are also accepted as markers of hepatic degeneration and dysfunction, but these are also associated with diabetes mellitus and metabolic syndrome [60]. ALT is also projected as an indicator of carotid atherosclerosis [61]. Therefore, we believe that an animal model offering abnormality in these biomarkers can serve as a worthy tool for CVD research.

We found that throughout the course of six weeks, the AST/ALT ratio remained unchanged and inferred the indication of acute and mild hepatic damage. Localization of AST is not confined to liver and may also be released on injury to heart or skeletal muscles [60]. Within hepatocytes, ALT is present in the cytoplasmic space and is released even on minor hepatic damage. AST, on the other hand, resides predominantly in mitochondria, and is discharged when the destruction is severe [62]. Therefore, an increase in the AST/ALT ratio would be observed with persistent liver damage.

Among the biomarkers of vascular function, we tried looking into uric acid (UA), phosphorus and ALP. We found these to be profoundly elevated by CCB-diet. ALP is considered as a potential diagnostic marker of CVD [63]. Apart from being involved in lipid absorption [64], ALP is also recognized to regulate vascular calcification [65–67]. Both ALP and UA correlate with hypertension [68, 69] and dyslipidemia [70, 71]. By facilitating smooth muscle cell proliferation [72], UA induces dysfunction of vascular endothelium [73], and hence acknowledged as a risk factor for atherosclerotic diseases [74]. Phosphorus impairs endothelial function [75] by prompting vascular calcification [76, 77] analogous to ALP, and is therefore, accredited as a amendable risk factor for atherosclerosis [78]. The reason we fail to achieve hyperglycemia at sixth week could be ascribed to phosphorus which enhances glucose utilization through glycolysis [79].

Noteworthy elevations in vascular biomarkers compelled us to explore the reactivity in thoracic aortae. As anticipated, aortae isolated from CCB-fed rats were evidently less responsive to ACh, indicative of endothelial dysfunction. We opted to further inquire, among the countless possibilities, the underlying nitric oxide pathway. Since the prior experiments deduced a probable malfunctioning endothelium, we examined endothelial-NOS enzyme activity, and found it to be compromised; with a consequent reduction in nitric oxide concentration. Revealing one of the precise underlying mechanisms, we aided future research by enabling scientists to confidently select and pin-point the mechanism of novel therapeutic interventions.

Apart from hepatic and vascular dysfunction, common CVD comorbidities also include memory impairment. Different HFDs are reported to delay the learning ability in experimental animals [80]. Therefore, we considered the likely consequence of this CCB-diet on rats’ memory and found consistent results, when CCB hindered the learning ability. Likewise, the fasting-induced prevention of memory-impairment that we acquired both in ND and CCB groups was also in accordance with the previous literature [81], where dietary restriction benefits learning capabilities [82].

## Conclusion

This study introduced the CCB-diet for multi-organ dysfunction in rats (a brief summary presented in Additional file 1), and proposed its use for research on cardiovascular diseases and associated manifestations. Like other commercially available expensive high-fat diets, this simple and robust CCB-diet induces hyperlipidemia in rats, which we showed, can be potentiated by coupling with two-hour fasting (daily). Further, we showed that this diet also offers elevation of biomarkers indicative of hepatic damage. Vascular function was simultaneously impaired, which we demonstrated at three levels; a) elevated vascular biomarkers, b) reduced endothelial reactivity of aorta and c) inhibition of nitric oxide pathway. Interestingly, CCB-diet also presented diminished memory/learning ability in rats. Hence, we suggest that the multi-organ abnormalities obtainable by this dietary model should be opted for research while inspecting the holistic effects of pharmaceutical interventions, specifically in complex disorders like cardiovascular diseases, where there is an immense interplay of integrated pathways.

## Methods and study design

### Animals and diets

Adult Sprague–Dawley rats (180 to 200 grams) of either gender were housed at the animal house of The Aga Khan University maintained at 23 to 25°C. These animals were kept in plastic cages with sawdust, and had free access to food and water (except for the fasting groups). The experiments conducted were in accordance with the guidelines for care and use of laboratory animals provided by The National Research Council [83]. The study protocol was approved by the Ethical Committee for Animal Care and Use, of The Aga Khan University, Karachi, Pakistan. Four groups of seven rats each were used. Group 1 (ND) and group 2 (ND-F) were fed with normal rat diets, whereas group 3 (CCB) and group 4 (CCB-F) were provided with cholesterol-cholate-butterfat diet [84]. Contents of both theses diets are given in Table 1. From group 2 (ND-F) and group 4 (CCB-F), food was withdrawn daily for two hours, whereas group 1 (ND) and group 3 (CCB) had *ad-libitum* access to food. At the end of the fourth week, blood was drawn from rats’ tail by cuff method [85]. However at the end of sixth week, blood was drawn through cardiac puncture.

**Table 1:** **Contents of normal and cholesterol-cholate-butterfat (CCB) diet**

Ingredients	Normal diet	CCB diet
Wheat flour	33.3%	30.9%
Bran fiber (choaker)	33%	30.6%
Fish meal	15%	13.9%
Dry skimmed milk powder	13.3%	12.3%
Cooking oil (soya)	3.3%	3.1%
Potassium metabisulphate	0.1%	0.1%
Salt	0.5%	0.5%
Nutrivet powder (bromix F-A)	0.33%	0.3%
Molasses	1%	0.9%
Cholesterol	–	2%
Cholic acid	–	0.5%
Butter fat	–	5%

### Learning ability or memory

In the sixth week rat’s learning ability or memory was assessed through Morris Water Maze (MWM) following the standard protocol [86] with slight modifications [87]. Briefly, in each trial, rats were allowed to swim in water and the time required to escape to the hidden platform, called escape latency, was recorded. This was continued for seven days such that the rats went through two trials on the first day and one trial per day for remaining six days.

### Biochemical estimations and enzyme assay

From the blood obtained on fourth and sixth week, serum was separated by centrifuging at 4000 rpm and 4°C for 10 min. The concentrations of total cholesterol (TC), triglyceride (TG), low-density lipoprotein (LDL), high-density lipoprotein (HDL), glucose (Gluc), gamma-glutamyl transferase (GGT), aspartate amino-transferase (AST), alanine amino-transferase (ALT), uric acid (UA), phosphorus (phosp.) and alkaline phosphatase (ALP) were estimated on Automated Analyzer (Roche Cobas c-111) using commercially available kits. Atherogenic index (AI) was calculated as the ratio of Non-HDL and HDL [21]. TC/HDL, LDL/HDL and AST/ALT ratios were also calculated. Serum Nitric oxide concentration was estimated by Griess method [88]. At the end of experiment (sixth week), thoracic aortae were isolated and endothelial nitric oxide synthase (eNOS) enzyme activity was assayed using the Nitric Oxide Synthase Assay Kit, Colorimetric (Calbiochem, Cat. No. 482702) following the manufacturer’s instructions.

### Vascular reactivity

On the aortae isolated from the rats of each group, the vascular reactivity was also assayed following the protocol of Furchgott and Zawadski [89] with certain modifications [90]. Briefly, aortic rings were mounted on the tissue bath and after acclimatization, concentration-response curves of acetylcholine (ACh: 0.01 μM to 100 μM) were prepared after pre-contracting the aortae with phenylephrine (PE: 1 × 10^−6^ mol/L).

### Statistical analysis

The data are expressed as mean ± SEM (Standard Error of Mean). For comparison between means of two groups, unpaired student's *t*-test was used. One-way analysis of variance (one-way ANOVA) was also applied when comparing the differences in means of four groups, followed by Tukey’s multiple comparison test to determine the significant differences. Two-way ANOVA followed by Bonferroni’s post-test was applied in vascular reactivity experiment to calculate the statistical significance. P-value less than 0.05 (p < 0.05) was considered as significant. Statistical analysis and plotting of graphs was done using GraphPad Prism software (version 4.0).

## Electronic supplementary material

Additional file 1: **Graphical Abstract showing cholesterol-cholate-butterfat (CCB) diet-induced increase in triglyceride (TG); total cholesterol (TC); low-density lipoprotein (LDL); atherogenic index (AI); ratio of TC/HDL; Ratio of LDL/HDL; glucose (Gluc); gamma-glutamul transferase (GGT); aspartate aminotransferase (AST); alanine aminotransferase (ALT); Ratio of AST/ALT; uric acid (UA); phosphorus (Phosp.) and alkaline phosphatase (ALP); loss of vascular endothelial reactivity, inhibition of nitric oxide (NO) production and endothelial nitric oxide synthase (eNOS) activity and memory impairment.** (JPEG 106 KB)

## Abbreviations

ACh: Acetylcholine
AI: Atherogenic index
ALP: Alkaline phosphatase
ALT: Alanine aminotransferase
AST: Aspartate aminotransferase
AST/ALT: Ratio of AST and ALT
CCB: Cholesterol-cholate-butterfat
CCB-F: Cholesterol-cholate-butterfat with fasting
CVD: Cardiovascular disease
eNOS: Endothelial nitric oxide synthase
GGT: Gamma-glutamyl transferase
HDL: High-density lipoprotein
LDL: Low-density lipoprotein
LDL/HDL: Ratio of LDL and HDL
MWM: Morris water maze
ND: Normal diet
ND-F: Normal diet fasting
NO: Nitric oxide
PE: Phenylephrine
Phosp: Phosphorus
TC: Total cholesterol
TC/HDL: Ratio of TC and HDL
TG: Triglyceride
UA: Uric acid
VLDL: Very-low-density lipoprotein.

## References

[1] Deaton C, Froelicher ES, Wu LH, Ho C, Shishani K, Jaarsma T (2011). The global burden of cardiovascular disease. Eur J Cardiovasc Nurs.

[2] Marchesini G, Brizi M, Bianchi G, Tomassetti S, Bugianesi E, Lenzi M, McCullough AJ, Natale S, Forlani G, Melchionda N (2001). Nonalcoholic fatty liver disease a feature of the metabolic syndrome. Diabetes.

[3] Montani J-P, Carroll JF, Dwyer TM, Antic V, Yang Z, Dulloo AG (2004). Ectopic fat storage in heart, blood vessels and kidneys in the pathogenesis of cardiovascular diseases. Int J Obes.

[4] Luchsinger JA, Mayeux R (2004). Cardiovascular risk factors and Alzheimer's disease. Curr Atherosclerosis Reports.

[5] Perseghin G (2010). The role of non-alcoholic fatty liver disease in cardiovascular disease. Digestive Disease.

[6] Weiner DE, Tabatabai S, Tighiouart H, Elsayed E, Bansal N, Griffith J, Salem DN, Levey AS, Sarnak MJ (2006). Cardiovascular outcomes and all-cause mortality: exploring the interaction between CKD and cardiovascular disease. Am J Kidney Dis.

[7] Haring B, Leng X, Robinson J, Johnson KC, Jackson RD, Beyth R, Wactawski Wende J, von Ballmoos MW, Goveas JS, Kuller LH (2013). Cardiovascular disease and cognitive decline in postmenopausal women: results from the Women's Health Initiative Memory Study. J Am Heart Assoc.

[8] Stampfer MJ (2006). Cardiovascular disease and Alzheimer's disease: common links. J Intern Med.

[9] Liao R, Carles M, Gwathmey JK (1997). Animal models of cardiovascular disease for pharmacologic drug development and testing: appropriateness of comparison to the human disease state and pharmacotherapeutics. Am J Ther.

[10] Herrera VL, Ruiz-Opazo N (2005). Genetic studies in rat models: insights into cardiovascular disease. Curr Opin Lipidol.

[11] Barrett G, Mullins JJ (1992). Transgenic animal models of cardiovascular disease. Curr Opin Biotechnol.

[12] Warden CH, Fisler JS (2008). Comparisons of diets used in animal models of high-fat feeding. Cell Metab.

[13] Goldsmith GA (1958). Dietary fat and human health: current recommendations. Am J Clin Nutr.

[14] Park S, Park Y (2009). Effects of dietary fish oil and trans fat on rat aorta histopathology and cardiovascular risk markers. Nutr Res Pract.

[15] Vles RO, Bueller J, Gottenbos J, Thomasson HJ (1964). Influence of Type of Dietary Fat on Cholesterol-Induced Atherosclerosis in the Rabbit. J Atheroscler Res.

[16] McAteer (2000). Effects of dietary fat and cholesterol on lipoprotein metabolism and on the development of atherosclerosis in hamster.

[17] Wicks MS, Ball CR, Williams WL (1969). Relation of types of dietary fat to cardiovascular damage in mice. Am J Anat.

[18] Hegsted DM, Andrus SB, Gotsis A, Portman OW (1957). The quantitative effects of cholesterol, cholic acid and type of fat on serum cholesterol and vascular sudanophilia in the rat. J Nutr.

[19] Akiyama T, Tachibana I, Shirohara H, Watanabe N, Otsuki M (1996). High-fat hypercaloric diet induces obesity, glucose intolerance and hyperlipidemia in normal adult male Wistar rat. Diabetes Res Clin Pract.

[20] McDonald SD, Pesarchuk E, Don-Wauchope A, El Zimaity H, Holloway AC (2011). Adverse metabolic effects of a hypercaloric, high-fat diet in rodents precede observable changes in body weight. Nutr Res.

[21] Siddiqi HS, Mehmood MH, Rehman NU, Gilani AH (2012). Studies on the antihypertensive and antidyslipidemic activities of Viola odorata leaves extract. Lipids Health Dis.

[22] Al-Attar AM (2010). Physiological and biochemical alterations in Sprague–Dawley female rats subjected to high fat diet and intermittent fasting. J Appl Sci Res.

[23] Gotto AM, Whitney E, Stein EA, Shapiro DR, Clearfield M, Weis S, Jou JY, Langendorfer A, Beere PA, Watson DJ, Downs JR, Cani JS (2000). Relation between baseline and on-treatment lipid parameters and first acute major coronary events in the Air Force/Texas Coronary Atherosclerosis Prevention Study (AFCAPS/TexCAPS). Circulation.

[24] Kannel WB (2005). Risk stratification of dyslipidemia: insights from the Framingham Study. Curr Med Chem Cardiovasc Hematol Agents.

[25] Manninen V, Tenkanen L, Koskinen P, Huttunen JK, Manttari M, Heinonen OP, Frick MH (1992). Joint effects of serum triglyceride and LDL cholesterol and HDL cholesterol concentrations on coronary heart disease risk in the Helsinki Heart Study. Implications for treatment. Circulation.

[26] Natarajan S, Glick H, Criqui M, Horowitz D, Lipsitz SR, Kinosian B (2003). Cholesterol measures to identify and treat individuals at risk for coronary heart disease. Am J Prev Med.

[27] Lemieux I, Lamarche B, Couillard C, Pascot A, Cantin B, Bergeron J, Dagenais GR, Despres JP (2001). Total cholesterol/HDL cholesterol ratio vs LDL cholesterol/HDL cholesterol ratio as indices of ischemic heart disease risk in men: the Quebec Cardiovascular Study. Arch Intern Med.

[28] Fernandez ML, Webb D (2008). The LDL to HDL cholesterol ratio as a valuable tool to evaluate coronary heart disease risk. J Am Coll Nutr.

[29] Yamamoto M, Yamamoto I, Tanaka Y, Ontko JA (1987). Fatty acid metabolism and lipid secretion by perfused livers from rats fed laboratory stock and sucrose-rich diets. J Lipid Res.

[30] Ide T, Ontko JA (1981). Increased secretion of very low density lipoprotein triglyceride following inhibition of long chain fatty acid oxidation in isolated rat liver. J Biol Chem.

[31] Heimberg M, Olubadewo JO, Wilcox HG (1985). Plasma lipoproteins and regulation of hepatic metabolism of fatty acids in altered thyroid states. Endocr Rev.

[32] Fungwe TV, Cagen LM, Cook GA, Wilcox HG, Heimberg M (1993). Dietary cholesterol stimulates hepatic biosynthesis of triglyceride and reduces oxidation of fatty acids in the rat. J Lipid Res.

[33] Ness GC, Zhao Z, Lopez D (1996). Inhibitors of cholesterol biosynthesis increase hepatic low-density lipoprotein receptor protein degradation. Arch Biochem Biophys.

[34] Cole TG, Kuisk I, Patsch W, Schonfeld G (1984). Effects of high cholesterol diets on rat plasma lipoproteins and lipoprotein-cell interactions. J Lipid Res.

[35] McNamara DJ (2002). Eggs and heart disease risk: perpetuating the misperception. Am J Clin Nutr.

[36] Boone LR, Brooks PA, Niesen MI, Ness GC (2011). Mechanism of resistance to dietary cholesterol. J Lipids.

[37] Ness GC, Gertz KR (2004). Hepatic HMG-CoA reductase expression and resistance to dietary cholesterol. Exp Biol Med (Maywood).

[38] Wang YM, Zhang B, Xue Y, Li ZJ, Wang JF, Xue CH, Yanagita T (2011). The mechanism of dietary cholesterol effects on lipids metabolism in rats. Lipids Health Dis.

[39] Sehayek E, Ono JG, Shefer S, Nguyen LB, Wang N, Batta AK, Salen G, Smith JD, Tall AR, Breslow JL (1998). Biliary cholesterol excretion: a novel mechanism that regulates dietary cholesterol absorption. Proc Natl Acad Sci U S A.

[40] Goh EH, Heimberg M (1977). Effects of free fatty acids on activity of hepatic microsomal 3-hydroxy-3-methylglutaryl coenzyme A reductase and on secretion of triglyceride and cholesterol by liver. J Biol Chem.

[41] Salam WH, Wilcox HG, Cagen LM, Heimberg M (1989). Stimulation of hepatic cholesterol biosynthesis by fatty acids. Effects of oleate on cytoplasmic acetoacetyl-CoA thiolase, acetoacetyl-CoA synthetase and hydroxymethylglutaryl-CoA synthase. Biochem J.

[42] Reynier MO, Montet JC, Gerolami A, Marteau C, Crotte C, Montet AM, Mathieu S (1981). Comparative effects of cholic, chenodeoxycholic, and ursodeoxycholic acids on micellar solubilization and intestinal absorption of cholesterol. J Lipid Res.

[43] Chen W, Suruga K, Nishimura N, Gouda T, Lam VN, Yokogoshi H (2005). Comparative regulation of major enzymes in the bile acid biosynthesis pathway by cholesterol, cholate and taurine in mice and rats. Life Sci.

[44] Maughan RJ, Fallah J, Coyle EF (2010). The effects of fasting on metabolism and performance. Br J Sports Med.

[45] Kersten S, Seydoux J, Peters JM, Gonzalez FJ, Desvergne B, Wahli W (1999). Peroxisome proliferator-activated receptor alpha mediates the adaptive response to fasting. J Clin Invest.

[46] Moller L, Stodkilde-Jorgensen H, Jensen FT, Jorgensen JO (2008). Fasting in healthy subjects is associated with intrahepatic accumulation of lipids as assessed by 1H-magnetic resonance spectroscopy. Clin Sci (Lond).

[47] Fukuda N, Ontko JA (1984). Interactions between fatty acid synthesis, oxidation, and esterification in the production of triglyceride-rich lipoproteins by the liver. J Lipid Res.

[48] Gibbons GF, Burnham FJ (1991). Effect of nutritional state on the utilization of fatty acids for hepatitic triacylglycerol synthesis and secretion as very-low-density lipoprotein. Biochem J.

[49] McGarry JD, Foster DW (1980). Regulation of hepatic fatty acid oxidation and ketone body production. Annu Rev Biochem.

[50] Meisinger C, Doring A, Schneider A, Lowel H (2006). Serum gamma-glutamyltransferase is a predictor of incident coronary events in apparently healthy men from the general population. Atherosclerosis.

[51] Dichi JB, Barbosa DS, Cecchini R, Dichi I (2008). Influence of uric acid and gamma-glutamyltransferase on total antioxidant capacity and oxidative stress in patients with metabolic syndrome. Nutrition.

[52] Packard CJ, Ford I, Robertson M, Shepherd J, Blauw GJ, Murphy MB, Bollen EL, Buckley BM, Cobbe SM, Gaw A, Hyland M, Jukema JW, Kamper AM, Macfarlane PW, Perry IJ, Stott DJ, Sweeney BJ, Twomey C, Westendorp RG (2005). Plasma lipoproteins and apolipoproteins as predictors of cardiovascular risk and treatment benefit in the PROspective Study of Pravastatin in the Elderly at Risk (PROSPER). Circulation.

[53] Lee DH, Jacobs DR, Gross M, Kiefe CI, Roseman J, Lewis CE, Steffes M (2003). Gamma-glutamyltransferase is a predictor of incident diabetes and hypertension: the Coronary Artery Risk Development in Young Adults (CARDIA) Study. Clin Chem.

[54] Webber M, Krishnan A, Thomas NG, Cheung BM (2010). Association between serum alkaline phosphatase and C-reactive protein in the United States National Health and Nutrition Examination Survey 2005–2006. Clin Chem Lab Med.

[55] Lippi G, Montagnana M, Franchini M, Favaloro EJ, Targher G (2008). The paradoxical relationship between serum uric acid and cardiovascular disease. Clin Chim Acta.

[56] Biasucci LM, Della Bona R, Cosentino N, Niccoli G, Minelli S, Gustapane M, Cialdella P, Bellone F, Basile E, Biasillo G (2011). Serum levels of gamma-glutamyltransferase predict coronary atherosclerosis progression in patients with ischemic heart disease under optimal medical therapy. J Am Coll Cardiol.

[57] Emdin M, Pompella A, Paolicchi A (2005). Gamma-Glutamyltransferase, Atherosclerosis, and Cardiovascular Disease Triggering Oxidative Stress Within the Plaque. Circulation.

[58] Franzini M, Corti A, Martinelli B, Del Corso A, Emdin M, Parenti GF, Glauber M, Pompella A, Paolicchi A (2009). Gamma-Glutamyltransferase activity in human atherosclerotic plaques - Biochemical similarities with the circulating enzyme. Atherosclerosis.

[59] Paolicchi A, Emdin M, Passino C, Lorenzini E, Titta F, Marchi S, Malvaldi G, Pompella A (2006). Beta-lipoprotein- and LDL-associated serum gamma-glutamyltransferase in patients with coronary atherosclerosis. Atherosclerosis.

[60] Goessling W, Massaro JM, Vasan RS, D'Agostino RB, Ellison RC, Fox CS (2008). Aminotransferase levels and 20-year risk of metabolic syndrome, diabetes, and cardiovascular disease. Gastroenterology.

[61] Hanley AJ, Williams K, Festa A, Wagenknecht LE, D'Agostino RB, Haffner SM (2005). Liver markers and development of the metabolic syndrome: the insulin resistance atherosclerosis study. Diabetes.

[62] Wang CC, Lin SK, Tseng YF, Hsu CS, Tseng TC, Lin HH, Wang LY, Kao JH (2009). Elevation of serum aminotransferase activity increases risk of carotid atherosclerosis in patients with non-alcoholic fatty liver disease. J Gastroenterol Hepatol.

[63] Tonelli M, Sacks F, Pfeffer M, Gao Z, Curhan G (2005). Relation between serum phosphate level and cardiovascular event rate in people with coronary disease. Circulation.

[64] Domar U, Karpe F, Hamsten A, Stigbrand T, Olivecrona T (1993). Human intestinal alkaline phosphatase release to the blood is linked to lipid absorption, but removal from the blood is not linked to lipoprotein clearance. Eur J Clin Investig.

[65] Limas CJ, Cohn JN (1973). Alkaline phosphatase in vascular smooth muscle. Nat New Biol.

[66] Lomashvili KA, Garg P, Narisawa S, Millan JL, O'Neill WC (2008). Upregulation of alkaline phosphatase and pyrophosphate hydrolysis: potential mechanism for uremic vascular calcification. Kidney Int.

[67] Narisawa S, Harmey D, Yadav MC, O'Neill WC, Hoylaerts MF, Millan JL (2007). Novel inhibitors of alkaline phosphatase suppress vascular smooth muscle cell calcification. J Bone Miner Res.

[68] Johnson RJ, Feig DI, Herrera-Acosta J, Kang DH (2005). Resurrection of uric acid as a causal risk factor in essential hypertension. Hypertension.

[69] Aliyu IS, Isah HS, Afonja OA (2006). Relationship between serum heat-stable alkaline phosphatase activity and blood pressure in patients with pre-eclampsia and eclampsia. Ann African Med.

[70] Keenan T, Blaha M, Nasir K, Silverman M, Carvalho J, Tota-Maharaj R, Conceicao R, Blumenthal R, Santos R (2012). Hyperuricemia predicts increased systemic inflammation, dyslipidemia and hepatic steatosis independent of obesity and metabolic syndrome. J Am Coll Cardiol.

[71] Ford ES, Li C, Cook S, Choi HK (2007). Serum concentrations of uric acid and the metabolic syndrome among US children and adolescents. Circulation.

[72] Corry DB, Eslami P, Yamamoto K, Nyby MD, Makino H, Tuck ML (2008). Uric acid stimulates vascular smooth muscle cell proliferation and oxidative stress via the vascular renin-angiotensin system. J Hypertens.

[73] Kanellis J, Kang DH (2005). Uric acid as a mediator of endothelial dysfunction, inflammation, and vascular disease. Semin Nephrol.

[74] Holme I, Aastveit AH, Hammar N, Jungner I, Walldius G (2009). Uric acid and risk of myocardial infarction, stroke and congestive heart failure in 417,734 men and women in the Apolipoprotein MOrtality RISk study (AMORIS). J Intern Med.

[75] Chen NX, D O'Neill K, Duan D, Moe SM (2002). Phosphorus and uremic serum up-regulate osteopontin expression in vascular smooth muscle cells. Kidney Int.

[76] Giachelli CM, Speer MY, Li X, Rajachar RM, Yang H (2005). Regulation of vascular calcification roles of phosphate and osteopontin. Circ Res.

[77] Jono S, McKee MD, Murry CE, Shioi A, Nishizawa Y, Mori K, Morii H, Giachelli CM (2000). Phosphate regulation of vascular smooth muscle cell calcification. Circ Res.

[78] Foley RN, Collins AJ, Herzog CA, Ishani A, Kalra PA (2009). Serum phosphorus levels associate with coronary atherosclerosis in young adults. J Am Soc Nephrol.

[79] DeFronzo RA, Lang R (1980). Hypophosphatemia and glucose intolerance: evidence for tissue insensitivity to insulin. N Engl J Med.

[80] Granholm AC, Bimonte-Nelson HA, Moore AB, Nelson ME, Freeman LR, Sambamurti K (2008). Effects of a saturated fat and high cholesterol diet on memory and hippocampal morphology in the middle-aged rat. J Alzheimers Dis.

[81] Pitsikas N, Algeri S (1992). Deterioration of spatial and nonspatial reference and working memory in aged rats: protective effect of life-long calorie restriction. Neurobiol Aging.

[82] Ingram DK, Weindruch R, Spangler EL, Freeman JR, Walford RL (1987). Dietary restriction benefits learning and motor performance of aged mice. J Gerontol.

[83] Institute of Laboratory Animal R (1996). Guide for the care and use of laboratory animals.

[84] Mandukhail SU, Aziz N, Gilani AH (2010). Studies on antidyslipidemic effects of Morinda citrifolia (Noni) fruit, leaves and root extracts. Lipids Health Dis.

[85] Furuhama K, Onodera T (1983). A simple technique for repeated blood collection from the tail vein of the rat. J Toxicol Sci.

[86] Morris R (1984). Developments of a water-maze procedure for studying spatial learning in the rat. J Neurosci Methods.

[87] Ahmed T, Gilani AH (2009). Inhibitory effect of curcuminoids on acetylcholinesterase activity and attenuation of scopolamine-induced amnesia may explain medicinal use of turmeric in Alzheimer's disease. Pharmacol Biochem Behav.

[88] Guevara I, Iwanejko J, Dembinska-Kiec A, Pankiewicz J, Wanat A, Anna P, Golabek I, Bartus S, Malczewska-Malec M, Szczudlik A (1998). Determination of nitrite/nitrate in human biological material by the simple Griess reaction. Clin Chim Acta.

[89] Furchgott RF, Zawadzki JV (1980). The obligatory role of endothelial cells in the relaxation of arterial smooth muscle by acetylcholine. Nature.

[90] Aziz N, Mehmood MH, Mandukhal SR, Bashir S, Raoof S, Gilani AH (2009). Antihypertensive, antioxidant, antidyslipidemic and endothelial modulating activities of a polyherbal formulation (POL-10). Vascul Pharmacol.

