# Supplementary figures and images for: Cholesterol-cholate-butterfat diet offers multi-organ dysfunction in rats
Source: Lipids Health Dis. 2014 Dec 16;13:194. doi: 10.1186/1476-511X-13-194 (PMC4290389; doi:10.1186/1476-511X-13-194)

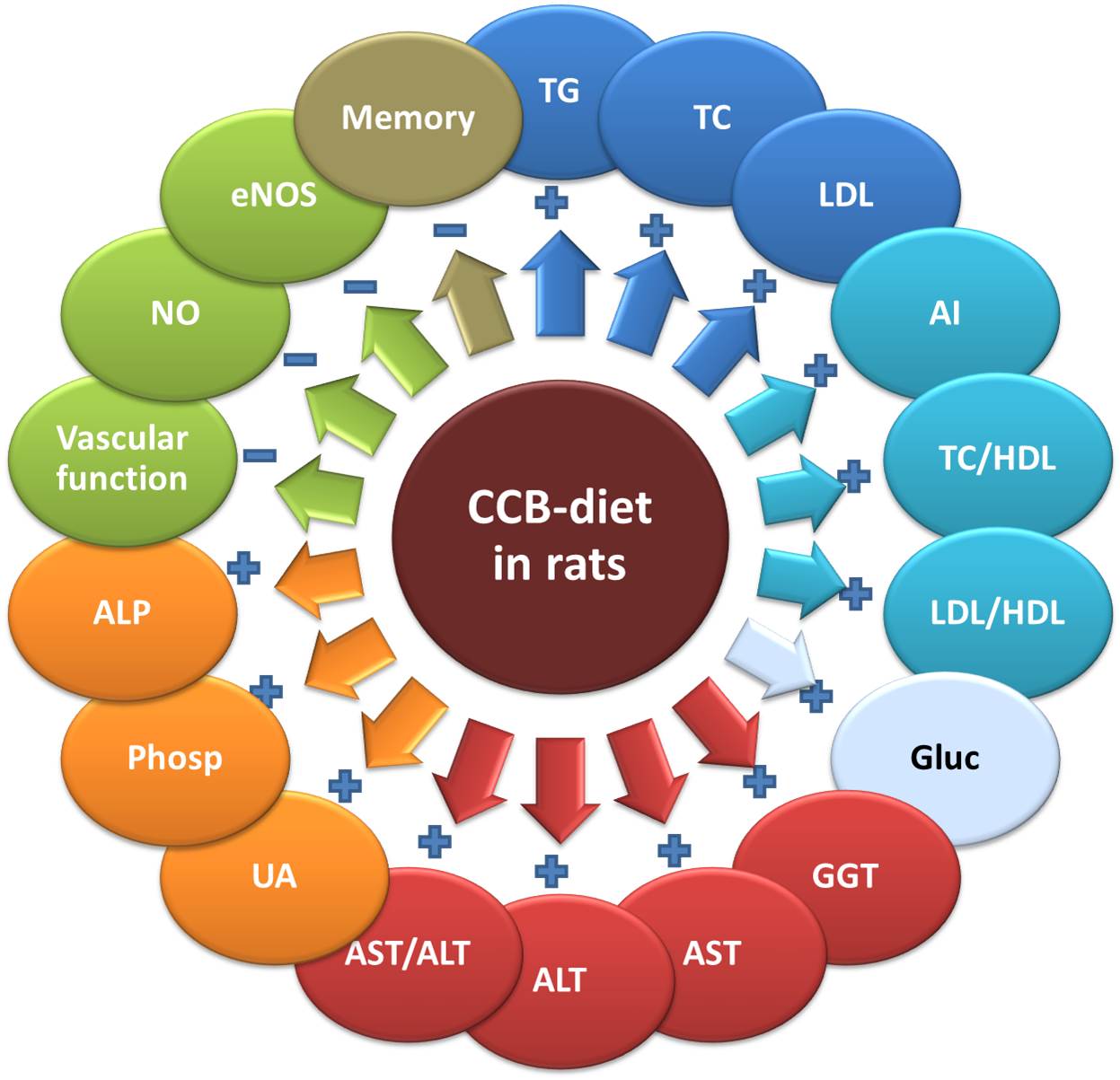

Supplement: Supplementary file 1 — Additional file 1: Graphical Abstract showing cholesterol-cholate-butterfat (CCB) diet-induced increase in triglyceride (TG); total cholesterol (TC); low-density lipoprotein (LDL); atherogenic index (AI); ratio of TC/HDL; Ratio of LDL/HDL; glucose (Gluc); gamma-glutamul transferase (GGT); aspartate aminotransferase (AST); alanine aminotransferase (ALT); Ratio of AST/ALT; uric acid (UA); phosphorus (Phosp.) and alkaline phosphatase (ALP); loss of vascular endothelial reactivity, inhibition of nitric oxide (NO) production and endothelial nitric oxide synthase (eNOS) activity and memory impairment. (JPEG 106 KB) [file 12944_2014_1178_MOESM1_ESM.jpeg]
